# Numerical Modeling of Micro-Mechanical Residual Stresses in Carbon–Epoxy Composites During the Curing Process

**DOI:** 10.3390/polym17121674

**Published:** 2025-06-17

**Authors:** Raffaele Verde, Alberto D’Amore, Luigi Grassia

**Affiliations:** Department of Engineering, University of Campania “Luigi Vanvitelli”, Via Roma 29, 81031 Aversa, Italy; raffaele.verde1@unicampania.it (R.V.); alberto.damore@unicampania.it (A.D.)

**Keywords:** polymer-based composites, curing, residual stresses, viscoelasticity, numerical simulation

## Abstract

This article analyzes the residual stresses generated during the curing process of thermoset composites. Specifically, a numerical procedure is developed and implemented in Ansys 18.0 to evaluate, at the micromechanical level, the residual stresses in a carbon epoxy composite that undergoes the process of curing. The viscoelastic behavior of the epoxy material is modeled using a formulation recently published by the same authors. It accounts for the concurrent effect of curing and structural relaxation on epoxy’s relaxation times, assuming thermo-rheological and thermo-chemical simplicities. The model validated for the neat epoxy matrix is now tested against the composite application. Various representative volume element (RVE) arrangements and fiber fractions are examined. The proposed procedure can predict the evolution of mechanical properties (apparent stiffness and creep compliance) and the residual stresses that develop in each composite constituent during the cure. It demonstrates that the residual stresses in the matrix are a consistent fraction of an epoxy’s nominal strength and significantly influence the transverse mechanical properties of the composite.

## 1. Introduction

Due to their excellent material properties in terms of high strength-to-weight and stiffness-to-weight ratios, polymer-based composites are extensively utilized in numerous engineering applications where high-performance and low-density materials are essential. Over the past few decades, the use of polymeric composites has significantly increased across various sectors, especially in aerospace [1,2].

However, a critical issue that restricts the application of composites is the scatter in mechanical properties induced by their manufacturing process. This occurrence leads to a more conservative design approach, requiring a large use of safety factor parameters, which diminishes their primary advantage of lightweight materials. During manufacturing, several defects can occur, such as voids, delamination [3], fiber debonding, warpage [4], part distortions [5], bent fibers, resin-deficient areas, resin-rich areas, and residual stresses. These defects reduce the theoretical mechanical properties of the composite, influence the structural performance, and sometimes cause problems during assembly due to out-of-tolerance distortions induced by the manufacturing process.

In this work, the attention is focused on the residual stresses that arise during the curing process and the factors that influence them. These stresses in the matrix phase can be a significant portion of the matrix’s nominal strength, and sometimes, the matrix can be locally damaged even before applying any external structural loads. Furthermore, matrix damage is often the precursor of several other degradation mechanisms (such as delamination or fiber failures) when the composite is subjected to fatigue [6]. Consequently, the presence of residual stresses can significantly reduce the fatigue life of the composite structure.

Given these considerations, it is clear that studying and quantifying the residual stresses resulting from manufacturing is crucial to improve the composite part quality and control the processing factors that influence these stresses.

Residual stresses in composite materials can be measured experimentally [7,8] or predicted numerically. The experimental techniques can be categorized into two types: those that directly measure the stresses and strains during manufacturing and those that evaluate the stresses after manufacturing. The first ones are based on applying external sensors, such as strain gauges and optical fibers, that monitor the evolution of stress and strain over time [9,10]. However, these techniques have limitations related to the sensor’s temperature sensitivity and their potential debonding—in particular, at low degrees of conversion [11].

Post-processing techniques are classified as non-destructive or destructive [7]. Destructive methods require the component’s rupture, and the most important ones are the first ply failure, hole drilling, and layer removal of symmetrical laminates. These methods remove a portion of the material, and the resulting deformation is measured [12,13]. These techniques generally do not have a high accuracy.

Non-destructive techniques, on the other hand, permit the saving of the component but are applicable only in some instances and for specific materials. Generally, the amount of stress is obtained indirectly by measuring the properties of the composite constituents, such as the electrical conductance or the polarization state of the light in the photoelasticity case [14,15]. These methods have limitations related to the nature of the material’s constituents (for instance, the photoelasticity and the micro-Raman spectroscopy [16] can be used only for transparent thermosetting or amorphous materials, the electrical conductivity method requires the presence of conductive fibers.

All the previous experimental techniques have disadvantages and are often expensive and unpracticable. For these reasons, numerical simulation can be beneficial in predicting residual stresses, understanding the factors influencing them, and overcoming experimental techniques’ limitations. On this topic, many studies were performed, and various numerical models were proposed, but despite all these efforts, the accurate prediction of the residual stresses in the composite remains a challenging task due to the complexity of modeling the curing process [17,18,19,20,21].

In fact, plausible results can be obtained only by accurately modeling the physical phenomena that occur during the process and their mutual interconnections. For instance, the crosslinking reaction is a temperature-activated phenomenon and, simultaneously, an exothermal process that causes a temperature variation; the thermal and kinetic fields are reciprocally influenced [17,18,22]. Moreover, the curing reactions produce a molecular structure change affecting epoxy’s viscoelastic properties, glass transition temperature, and volume expansion coefficient [23].

The first step to achieve accurate simulations is to understand and model all the previous physical phenomena, considering their interconnections. Many studies have been conducted on this topic, mainly focusing on how crosslinking reactions influence the mechanical properties of thermosetting systems [24,25,26,27,28]. Several simplified models [19,20,21,29] assume an elastic behavior and use empirical relationships to correlate the elastic modulus with the temperature and the degree of conversion. Bogetti et al. [19] initially proposed an elastic formulation in which the epoxy modulus varies with the degree of conversion. The previous approach was later enhanced by including the effect of temperature as reported in the CHILE (Cure Hardening Instantaneous Linear Elastic) model [20]. Despite their simplicity in modeling and finite element implementation, these models fail to account for the effect of stress relaxation, leading to an overestimation of residual stresses.

Kim and White [27] developed a viscoelastic formulation. They carried out experiments by curing the epoxy at different temperatures to obtain different conversion degrees; these samples were tested in stress relaxation using DMA to evaluate the polymer’s master curves for each degree of conversion. From these results, they proposed an expression for the time–temperature–degree of the conversion shift factor.

During curing, internal stresses start to build up, mainly due to the presence of non-uniform free strain distributions within the volume. Free strain refers to material strains produced by volume changes induced by temperature variations, humidity adsorption, or molecular structure contractions due to crosslinking in thermosetting polymers or crystallization in semicrystalline ones. In a homogeneous body, the volume contractions do not produce a tensional state, but stresses start to build up when different points within the curing volume do not equal free strains at the same instant of the cure cycle. The presence of fibers with different thermal and mechanical properties compared to the polymeric matrix contributes to an increased accumulation of internal stresses [21].

Additionally, the orthotropic nature of composite laminae causes mismatches in the mechanical and thermal properties between adjacent layers in non-unidirectional laminates, leading to distortions and residual stresses in the laminate [5]. The interaction between the part and the tool and external constraints also influences the development of internal stresses. When these stresses reach critical levels, the composite may experience matrix damage or cracking in the matrix phase before applying any external loads.

Many studies have been conducted to understand the origins of residual stresses [17,20,30,31,32,33] in polymers and polymeric composites. In particular, D’Amore et al. [34,35] numerically investigated the residual damage induced in an amorphous polymer during the cooling phase from the melt to the glassy state. Their results showed that structural relaxation has a significant influence on the growth of internal stresses. Simon et al. [36] and Zarelli et al. [37,38] experimentally evaluated the residual stresses in constrained thermoset polymers, showing that the volume contraction due to crosslinking reactions and the subsequent thermal cycling can produce significant internal stresses.

In a more recent work [39], the authors, based on the Kim and White’s experimental data [27], modeled the combined influences of curing and structural relaxation on the viscoelastic behavior of epoxy and proposed a new expression for the shift factor based on the well-validated TNM (Tool–Narayanaswamy–Moynihan) [40] model. Based on the hypothesis of thermochemical and thermoreological simplicity, the formulation was then applied to simulate the curing process of an epoxy cylinder [41] subjected to a non-uniform thermal history. The residual stress can be a significant fraction of the resin’s nominal strength. They develop predominantly during cooling to ambient temperature and tend to increase with the Biot number.

Assessing residual stresses in fiber-reinforced composites is more difficult than in purely polymeric materials, primarily due to the complexity of modeling the interaction between the two phases. Several cure models have been proposed in the literature to estimate the residual stresses and evaluate their impact on the composite’s effective mechanical properties at the micro- and macroscale. For instance, Maiaru et al. [29] numerically investigated how the manufacturing process affects the transverse strength of unidirectional fiber-reinforced polymers at the microscale. In a more recent work [42], they predicted the residual stresses and the distortions in cross-ply laminates at the end of the curing cycle, proposing a higher-order finite element modeling approach.

However, many of these models assume an elastic behavior of the resins, neglecting the stress relaxation associated with the viscoelastic nature of polymers.

The objective of this work is to numerically predict the residual stresses in a carbon epoxy composite using a previously proposed viscoelastic formulation proposed by the same authors. Compared to other available models in the literature, this formulation allows to model the stress relaxation due to the viscoelastic nature of the polymer, accounting for the concurrent effect of curing and structural relaxation on the polymer’s relaxation times. The aim is to predict the internal stress evolution in a unidirectional carbon–epoxy composite at the micromechanical scale and assess how the manufacturing process influences the composite’s mechanical properties.

## 2. Materials and Methods

### 2.1. Mathematical Formulation

The mathematical formulation complexity needed to model the viscoelastic behavior of the resin during the curing [39] is summarized here. First, we start by modeling the reaction kinetics of a commercially available 3501-6 epoxy resin (Hercules, Inc., Wilmington, DE, USA) representing the matrix of the adopted composite representative volume element (to be discussed later).

The key parameter used to identify the progress of the curing process is the degree of conversion, defined as

(1) $$\alpha =\frac{H(t)}{H_{r}}$$

where *H*(*t*) is the reaction heat generated at time *t*, and *H_r_* is the total reaction heat released upon a complete cure. The rate of conversion depends on both temperature and time, and its evolution follows the kinetic model developed by Bogetti et al. [18]:

(2) $$\frac{d\alpha }{dt}=(k_{1}+k_{2}\;\alpha )\left(0.47-\alpha \right)\;\;\;\;\;\;\;\;\;for\;(\alpha \leq 0.3)$$

(3) $$\frac{d\alpha }{dt}=k_{3}\left(1-\alpha \right)\;\;\;\;\;\;\;\;for\;(\alpha >0.3)$$

*α* = 0.3 at the gel point, and *k*_1_, *k*_2_, and *k*_3_ follow the Arrhenius law:

(4) $$k_{i}=A_{i}\;Exp\left(-\frac{{\Delta E}_{i}}{RT}\right)\;\;\;\;\;i=1,2,3$$

where *A_i_* (*i* = 1, 2, 3) are material pre-exponential factors, *T* is the absolute temperature, *R* is the gas universal constant, and Δ*E_i_* are the activation energies.

The curing is an exothermic process, and the specific internal heat in a fiber composite material is proportional to the conversion rate and expressed as

(5) $$Q=\rho \;\frac{\rho }{\rho_{c}}\;{(1-V_{f})H}_{r}\frac{d\alpha }{dt}$$

where *H_r_* represents the total heat released per unit mass of epoxy, *α* is the degree of conversion, *V_f_* is the fiber volume fraction, *ρ* is the matrix density, and *ρ_c_* is the composite density. The reaction rate is thermally activated and is influenced by the heat released during the exothermic process. In the case under study, due to the tiny dimensions of the analyzed model, the temperature is considered uniform throughout the entire geometry, and the effects of internal heat generation from the exothermic process can be neglected.

The epoxy resin viscoelastic behavior can be modeled using the constitutive framework of linear viscoelasticity. Under this approach, the stress–strain relationship is formulated as follows:

(6) $$\sigma_{ij}=\int_{0}^{\xi }G(\alpha ,\xi -\xi^{\prime })\frac{de_{ij}}{d\xi^{\prime }}\;\;{d\xi }^{\prime }+\delta_{ij}\int_{0}^{\xi }\frac{1}{3}\;K(\alpha ,\xi -\xi^{\prime })\frac{d(\varepsilon -\varepsilon_{free})}{d\xi^{\prime }}\;\;{d\xi }^{\prime }$$

The term $\varepsilon_{free}$ represents the free strain induced by non-mechanical loads. In this case, it comprises both thermal and chemical strains. The chemical strain (shrinkage), resulting from the formation of the crosslinked network, is assumed to vary proportionally with the degree of conversion.

The shear and bulk relaxation moduli are denoted by *G* and *K*, respectively. The bulk modulus is treated as a constant in time, as its relaxation is negligible compared to the shear relaxation [43]. The shear modulus, instead, is a strong function of both temperature and degree of conversion. It is described through a Prony series expansion, as follows:

(7) $$G\left(\xi \right)=G_{0}\;\left[\alpha_{\infty }^{G}+\sum_{i=1}^{n_{G}}\alpha_{i}^{G}\exp\left(-\frac{\xi }{\tau_{i}^{G}}\right)\right]$$

*G*(*ξ*) is expressed by a series of Maxwell elements, each defined by a characteristic relaxation time, $\tau_{i}^{G}$, and a corresponding weight factor, $\alpha_{i}^{G}$.

$\alpha_{\infty }^{G}$ is defined as follows:

(8) $$\alpha_{\infty }^{G}=\frac{G_{0}-G_{\infty }}{G_{0}}$$

$G_{0}$ and $G_{\infty }$ are the unrelaxed (glassy) and the fully relaxed (rubbery) shear moduli. The rubbery modulus is assumed to be nearly zero and independent of the degree of curing [27].

*ξ* is the reduced time, expressed as

(9) $$\xi =\int_{0}^{t}\frac{1}{a(T,\alpha )}\;dt^{\prime }$$

$a\left(T,\alpha \right)$ is the shift factor considering the influence of temperature, degree of conversion, and structural relaxation on the epoxy relaxation times. Assuming the well-validated hypothesis of thermo-rheological and chemo-rheological simplicities (namely, changes in the degree of cure and the temperature do not alter the shape of viscoelastic functions but only shift them on their timescale) [26,44,45], the relaxation times, τ, at a generic temperature and degree of conversion state can be expressed as

(10) $$\tau \left(T,\alpha \right)=\frac{\tau_{R}}{a\left(T,\alpha \right)}$$

The reference relaxation time, $\tau_{R}$, is defined at the glass transition temperature, $T_{g}$, that is evaluated for each degree of conversion. It is assumed that, independently of the degree of the curing, the relaxation time remains the same at the glass transition temperature.

The evolution of $T_{g}$ with the degree of cure is described by a quadratic function [27]:

(11) $$T_{g}\left(\alpha \right)=b_{1}+b_{2}\;\alpha +b_{3}\;\alpha^{2}$$

where *b*_1_, *b*_2_, and *b*_3_ are material parameters.

Given the degree of cure, the shift factor is evaluated using a modified TNM equation [40], expressed as follows:

(12) $$a\left(T,\alpha ,T_{f}\right)=exp\left(-\frac{\Delta H\left(\alpha \right)}{R}\;\left(\frac{x\left(\alpha \right)}{T}+\frac{1-x\left(\alpha \right)}{T_{f}}-\frac{1}{T_{g}(\alpha )}\right)\right)$$

(13) $$\Delta H\left(\alpha \right)=k_{4}+k_{5}\alpha +k_{6}\alpha^{2}$$

(14) $$x\left(\alpha \right)=k_{7}+k_{8}\alpha$$

Δ*H* is the activation energy, and *x* is the non-linearity parameter partitioning the relaxation phenomena between the temperature and the corresponding fictive temperature, $T_{f}$. These parameters depend on the degree of curing, and k_4_, k_5_, k_6_, k_7_, and k_8_ are material constants. The fictive temperature, $T_{f}$ [46], is a variable that characterizes the structure of the glassy state. The time dependence of the fictive temperature can be described using the TNM model [40]:

(15) $$T_{f}\left(t\right)=T\left(t\right)-\int_{0}^{t}M(\xi \left(t\right)-\xi \left(t^{\prime }\right))\;\;\frac{dT(t^{\prime })}{dt^{\prime }}dt^{\prime }$$

where *ξ*(*t*) denotes the reduced time. According to Equation (12), it can be expressed as

(16) $$\xi \left(t\right)=\int_{0}^{t^{\prime }}exp\left[\frac{\Delta H\left(\alpha \right)}{R}\;\left(\frac{x\left(\alpha \right)}{T}+\frac{1-x\left(a\right)}{T_{f}}-\frac{1}{T_{g}(\alpha )}\right)\right]dt^{\prime }$$

The memory function *M*(*ξ*) is also expressed by a Prony series expansion:

(17) $$M\left(\xi \right)=\sum_{i=1}^{N_{m}}\alpha_{i}^{M}\;exp(-\frac{\xi }{\tau_{i}^{M}})$$

(18) $$\sum_{i=1}^{N_{m}}\alpha_{i}^{M}=1$$

Equation (17) assumes that the structural relaxation can be decomposed in $N_{m}$ relaxation modes; each has its relaxation time $\tau_{i}^{M}$, partial fictive temperature $T_{f_{i}}$, and weight factor $\alpha_{i}^{M}$. Partial fictive temperatures can be defined as

(19) $$\frac{dT_{f_{i}}}{dt}=-\frac{T_{f_{i}}-T}{{\tau_{i}}^{M}}\frac{d\xi }{dt}$$

and their weighted sum is the actual fictive temperature.

To solve the previous equations, the implicit and unconditionally stable numerical scheme developed by Markovsky and Soules [47] was adopted. The partial fictive temperature at a given time step *k*, $T_{f_{i}}\left(k\right)$, is calculated as

(20) $$T_{f_{i}}\left(k\right)=\frac{{\tau_{i}}^{M}\;T_{f_{i}}\left(k-1\right)+T\left(k\right)dt\;a(T_{f}(k-1))}{{\tau_{i}}^{M}+dt\;a(T_{f}(k-1))}$$

(21) $$T_{f}\left(k\right)=\sum_{i=1}^{N}\alpha_{i}^{M}\;T_{f_{i}}\left(k\right)$$

Moreover, since the proposed shift factor expression is not present in Ansys, the proposed model was implemented in Ansys through a modified Arrhenius model. In particular, the equality between the two expressions was imposed:

(22) $$exp\left(\frac{\Delta H\left(\alpha \right)}{R}\left(\frac{x(\alpha )}{T}+\frac{1-x(\alpha )}{T_{f}}-\frac{1}{T_{g}\left(\alpha \right)}\right)\right)=exp\left(\frac{{\Delta H}_{Arrhenius}}{R}\left(\frac{1}{T}-\frac{1}{T_{g}\left(\alpha \right)}\right)\right)$$

The “fictitious activation energy”, Δ*H_Arrhenius_*, is expressed as

(23) $${\Delta H}_{Arrhenius}(\alpha ,T_{f},T)=\frac{\Delta H\left(\alpha \right)\;T\;\left(T_{f}+T_{r}\left(-1+x\right)\right)-T_{g}(\alpha )\;x)}{T_{f}\;(T-T_{g}(a))}$$

### 2.2. Finite Elements Model (FEM) Description

**Figure 1:**
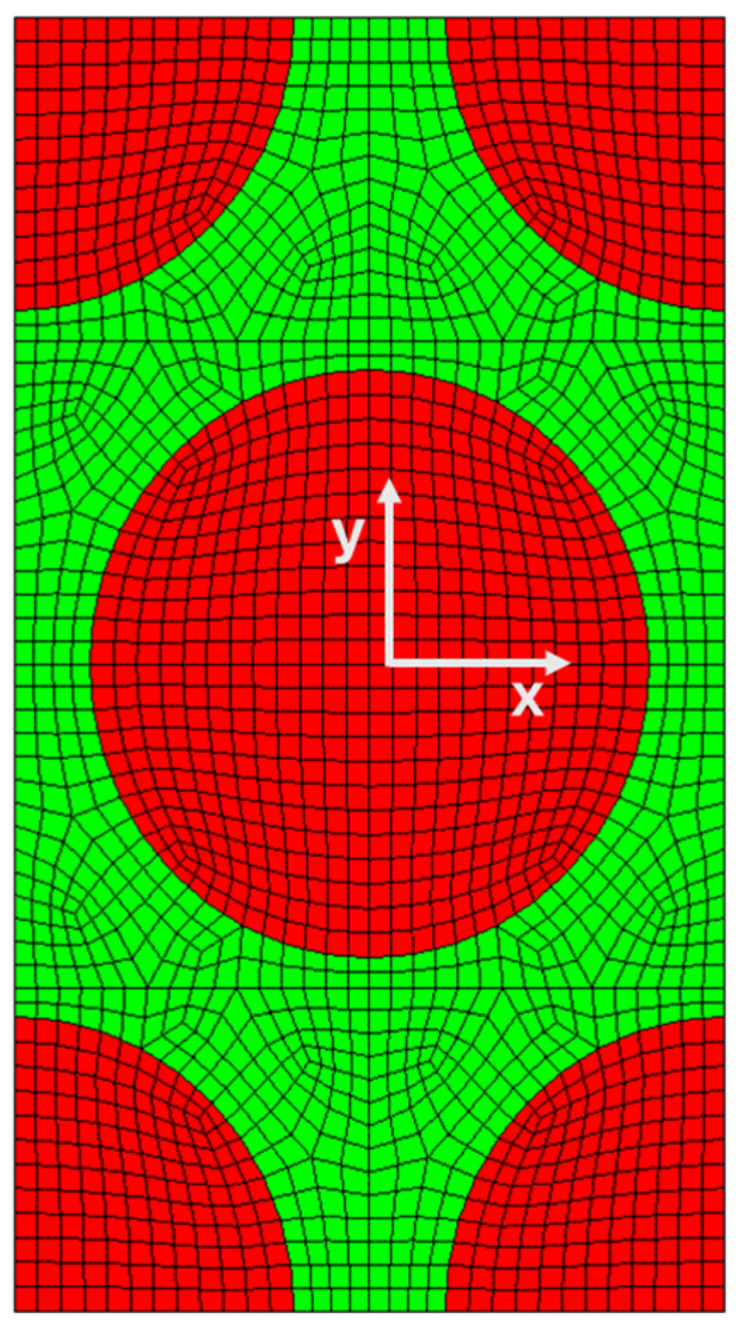
Representative volume element (RVE).

The analyzed geometry is a composite’s representative volume element (RVE) made of carbon fibers and an epoxy matrix. Different fiber fractions and RVE arrangements are analyzed. In the first scenario, the fiber’s volume fraction is considered to be 0.6, and the fibers are arranged in a hexagonal array, as shown in Figure 1. The dimensions of the RVE are 6.35 × 11.0 μm. The RVE is subdivided into 2500 elements with a characteristic dimension of 0.15 μm.

The considered carbon fibers are HexTow IM7 (Hexcel). They have a diameter of 5 μm and are modeled as linearly elastic materials with transversely isotropic behavior. Instead, the resin exhibits a linear viscoelastic behavior, modeled as Section 2.1 describes.

Since the composite shows much higher stiffness in the longitudinal direction of the fibers than in the transverse directions, plane elements with the plane strain option are utilized. Specifically, the PLANE 182 element from the Ansys library is adopted: it is a 4-node structural element with two degrees of freedom for each node (the two translations in the plane direction).

Conceptually, the RVE is the smallest unit that captures all the significant aspects of the material, and by repeating it, the entire composite can be constructed. Since, in reality, there are many other RVEs at its contour, periodic boundary conditions (PBCs) need to be applied. These conditions ensure continuity of displacement across the RVE’s boundaries and preserve the periodicity of the modeled system.

PBCs are postulated by Xia et al. [48] and used in many cases to model a pervasive heterogeneous system by only modeling a characteristic RVE. In the case under study, they can be expressed as follows:

(24) $$u_{x}\left(a_{x},y\right)-u_{x}\left(-a_{x},y\right)=\varepsilon_{x}\;l_{x}$$

(25) $$u_{y}(x,a_{y})-u_{y}(x,-a_{y})=\varepsilon_{y}\;l_{y}$$

(26) $$u_{y}\left(a_{x},y\right)-u_{y}\left(-a_{x},y\right)={2\;\varepsilon }_{xy}\;l_{x}$$

(27) $$u_{x}\left(x,a_{y}\right)-u_{x}\left(x,-a_{y}\right)={2\;\varepsilon }_{xy}\;l_{y}$$

where *l_x_* and *l_y_* are the RVE dimensions along the x and y directions, respectively. *a_x_* = *lx*/2, and *a_y_* = *ly*/2. *ε_x_*, *ε_y_*, and *ε_xy_* are the strains induced on the RVE by applying external loads. During the cure, they are due to thermal and chemical strains that occur during the process.

Periodic boundary conditions are implemented in Ansys APDL by applying constraints between the nodes on opposing sides of the RVE. Additionally, the central node of the RVE, located at the origin of the coordinate system, is constrained with zero displacements in both the x and y directions to prevent rigid body motion of the system.

**Figure 2:**
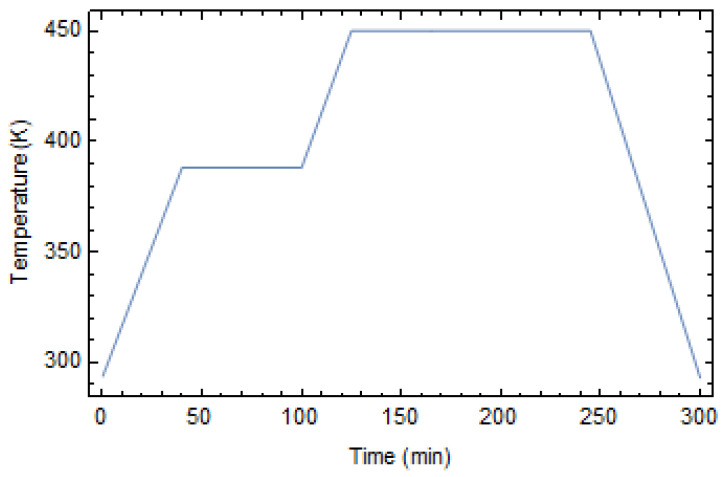
Curing thermal profile.

A two-step temperature profile, recommended for the epoxy used and plotted in Figure 2, was applied to the RVE. Initially, the system is heated from room temperature to 388 K and held at this temperature for 60 min. Then, the material is heated to the curing temperature Tcure = 450 K and maintained at this temperature for 120 min. Finally, the system is cooled to room temperature at a constant rate of 2.76 K/min. Since the geometrical volume is tiny, the temperature is considered uniform across the whole geometry.

A transient analysis is conducted in Ansys to simulate the curing process. Thermokinetics and structural analysis are coupled using the structural module of Ansys and a homemade routine written in APDL language. At each time step, the thermokinetic problem is first solved within the custom APDL routine. Based on the current temperature, the kinetic equations (Equations (2) and (3)) are integrated using the explicit Euler method, allowing for the calculation of the degree of conversion for that time step. Subsequently, using the computed degree of cure, the same routine evaluates the shift factor parameters (Equations (13) and (14)) and calculates the fictive temperature using the numerical algorithm proposed by Soules [47], as detailed in Section 2.1, allowing the determination of the epoxy’s viscoelastic properties. With the updated material properties, the structural problem is then solved using the structural module of Ansys. The entire procedure is iteratively repeated across all time steps to compute the evolution of the stresses and other material parameters during the curing process.

**Figure 3:**
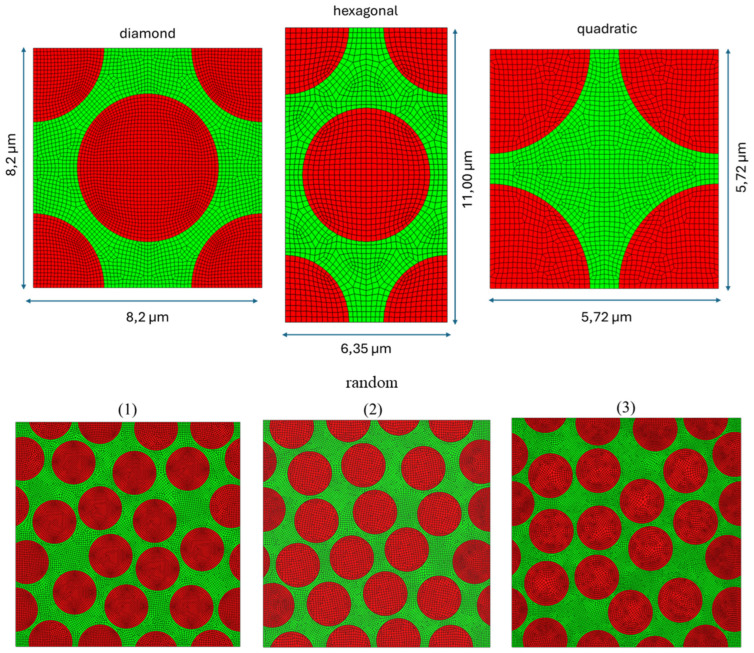
Different fibers distribution (fiber volume fraction V_f_ = 0.6).

Various fiber volume fractions and geometrical arrangements of fibers are examined to assess how the fraction of fibers and their distribution within the matrix influence the distribution of residual stresses. In particular, hexagonal, quadratic, and diamond arrays are modeled. Moreover, three random arrays are also considered, since the fibers are dispersed randomly in the resin. These random arrays have dimensions of 25.6 × 25.6 μm and consist of 20 carbon fibers with a total fiber fraction of 0.6. They are generated using a homemade routine in the software Mathematica 11.3. In all the RVEs, the fibers and the matrix are fully bonded, and no interface elements are modeled (the fibers and the matrix share common nodes at their interface). The discretized geometries are illustrated in Figure 3.

For all the analyzed models, quadrilateral elements were used to discretize the system. The mesh is fine enough to assure a good numerical accuracy of the results. A mesh sensitivity study was performed by refining the mesh (splitting each FEM element in 4 smaller elements), and no variation of the results was found.

Additionally, to assess the impact of curing-induced internal stresses on the mechanical properties of a composite, a virtual simulation of tensile, compression, and shear tests are conducted for both the cured-stressed and the ideally cured-unstressed configurations. Simulations are done to obtain stress–strain curves of the cured-stressed and the ideally initial unstressed cases for all the previous fiber arrangements.

### 2.3. Model Parameters

This article investigates the physical behavior of the 3501-6 epoxy resin (Hercules, Inc., Wilmington, DE, USA), used mainly in aerospace [27]. A precise description of all model parameters for this epoxy, including sources and related assumptions, is provided in a previous study [39]. Here, only a concise overview of the input data is provided.

The thermal and curing parameters, originally reported by Bogetti et al. [18] are summarized in Table 1.

The relaxation times of the epoxy are obtained from the work of Kim and White [27], and the experimental values of *T_g_* are deduced from the shift factors data. It is assumed that the fully cured epoxy’s glass transition temperature is 195 °C, and at the glass transition temperature, the relaxation time does not vary with the degree of conversion [39].

The viscoelastic response of the epoxy is characterized by assigning the instantaneous values of two viscoelastic functions and the Prony’s coefficients for the shear relaxation at the reference state (that is assumed to be at the glass transition temperature for each degree of conversion). The instantaneous mechanical response is defined by assuming an elastic modulus of 3.2 GPa and a Poisson ratio of 0.35 [27]. The Prony series parameters used in the shear relaxation modulus (Equation (7)) are listed in Table 2.

Material constants related to the glass transition temperature (*T_g_*), activation energy (Δ*H*), and the non-linearity parameter (*x*) used to compute the shift factor in Equation (12) are provided in Table 3.

The Prony coefficients for the memory function at the glass transition temperature are reported in Table 4.

The orthotropic mechanical properties of the fibers [29] are reported in Table 5. Direction 1 is the fiber direction, while directions 2 and 3 are the transverse directions. Since the fiber is transversally isotropic, the material properties in directions 2 and 3 are identical.

## 3. Results and Discussion

**Figure 4:**
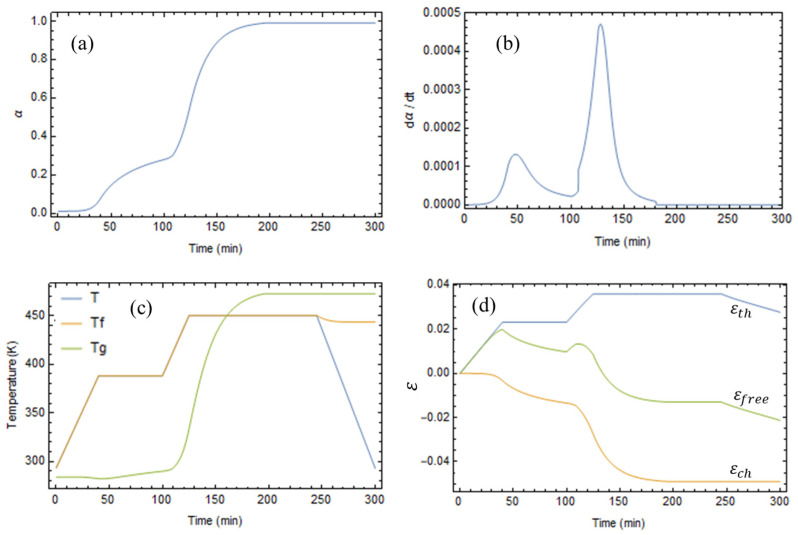
Evolution of the resin’s properties during the process: (**a**) degree of conversion, (**b**) rate of conversion, (**c**) glass transition temperature, and (**d**) free strains.

The degree of conversion evolves in time until reaching a unit value approximately at t = 200 min, as shown in Figure 4a. At this time, the curing reaction can be considered complete, and the degree of conversion stops evolving. Figure 4b illustrates the conversion rate during the process, showing that it increases during heating and decreases while the temperature is constant, since the cure is a temperature-activated phenomenon.

The evolution of the resin’s glass transition temperature is illustrated in Figure 4c. The curve labeled $T_{g}$ is the glass transition temperature according to the cooling conditions of the experimental data. The glass transition temperature depends on the polymer’s thermal history and may differ from the $T_{g}$. $T_{f}$ is the fictive temperature, which is equal to the local temperature in the rubbery state and matches the actual glass transition temperature in the glassy state. In the initial stages of the process, the fictive temperature coincides with the local temperature, and the material remains in its rubbery state until cooling to room temperature. At this point, the fictive temperature exceeds the local temperature, making the resin glassy.

Figure 4d reports the components of the free strains. Initially, the resin expands, because the positive thermal strain, *ε_th_*, induced by temperature variation is higher than the chemical strain, *ε_ch_*, associated with the crosslinking reaction. Then, the absolute value of the chemical strain exceeds the thermal strain, and at the end of the process, the resin has undergone a 2% contraction.

**Figure 5:**
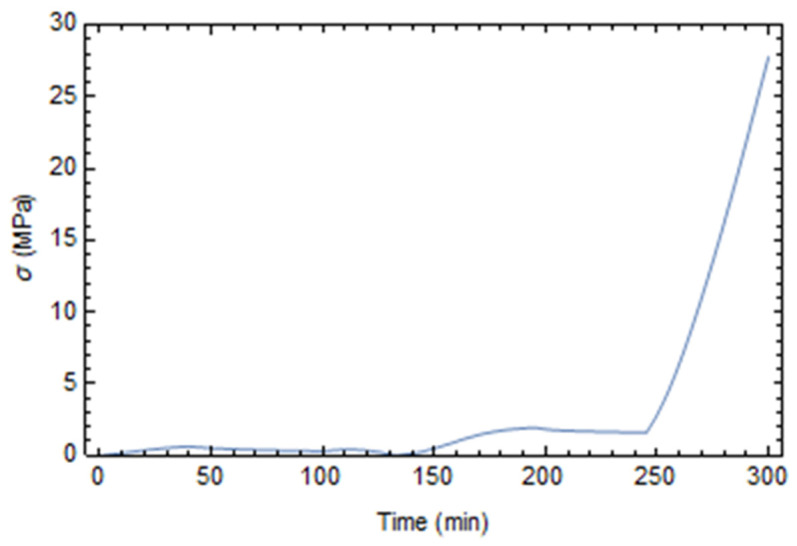
Maximum von Mises stress during the process.

The origin of residual stresses has to be searched regarding the significant mismatch in thermal and mechanical properties between the fibers and the matrix. Residual stresses develop significantly during the cooling phase, as shown in Figure 5. Before cooling, in fact, the residual stress is negligible, since the stress relaxation occurs almost instantly at high temperatures and a low degree of cure.

**Figure 6:**
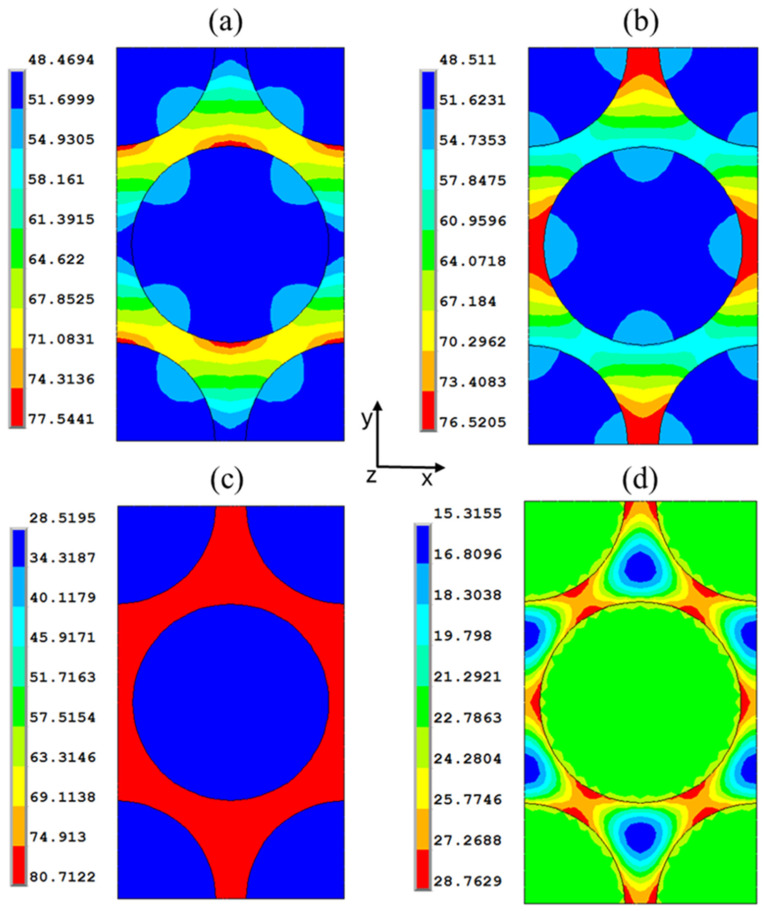
Components of residual stresses (MPa) in the hexagonal RVE. Fiber volume fraction V_f_ = 0.6: (**a**) x component of stress, (**b**) y component of stress, (**c**) z component of stress, and (**d**) von Mises stress.

Figure 6 reports the stress components and the von Mises stress contour plots at the end of the curing process. The stress levels are higher in the regions where the matrix is closer to the fibers. It is noted that the matrix undergoes a final tensile stress at the end of the curing. The amount of stress in the fibers at the end of the process is negligible compared to their nominal strength.

**Figure 7:**
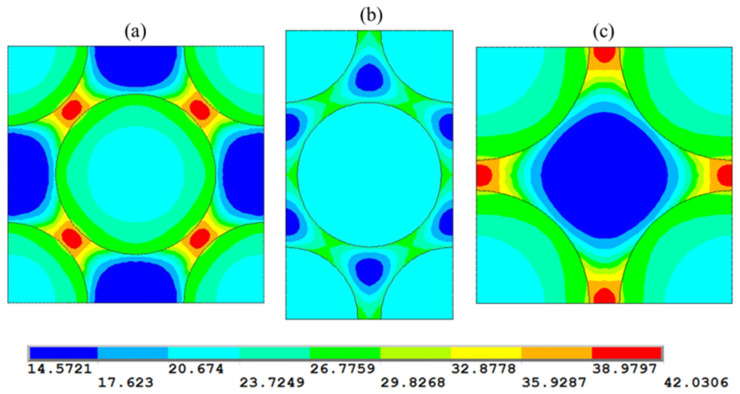
von Mises stress (MPa) contour plot in the three examined fiber arrangements: (**a**) diamond array, (**b**) hexagonal array, and (**c**) quadratic array. Fiber volume fraction V_f_ = 0.6.

Figure 7 reports the Von Mises contour plots for the three configurations. The results reveal that the fiber’s arrangement significantly affects the spatial distribution of stresses. In the quadratic and diamond arrays, the maximum stress in the matrix is higher than in the hexagonal one. The mean stress is calculated according to the following equation:

(28) $$\sigma_{M}=\frac{1}{V}\int \sigma \;dV$$

It was found that the mean stress of about 22 MPa is almost the same for all three configurations. Therefore, the fiber arrangement does not impact the mean stresses in the matrix phase but affects the spatial distribution of stress.

**Figure 8:**
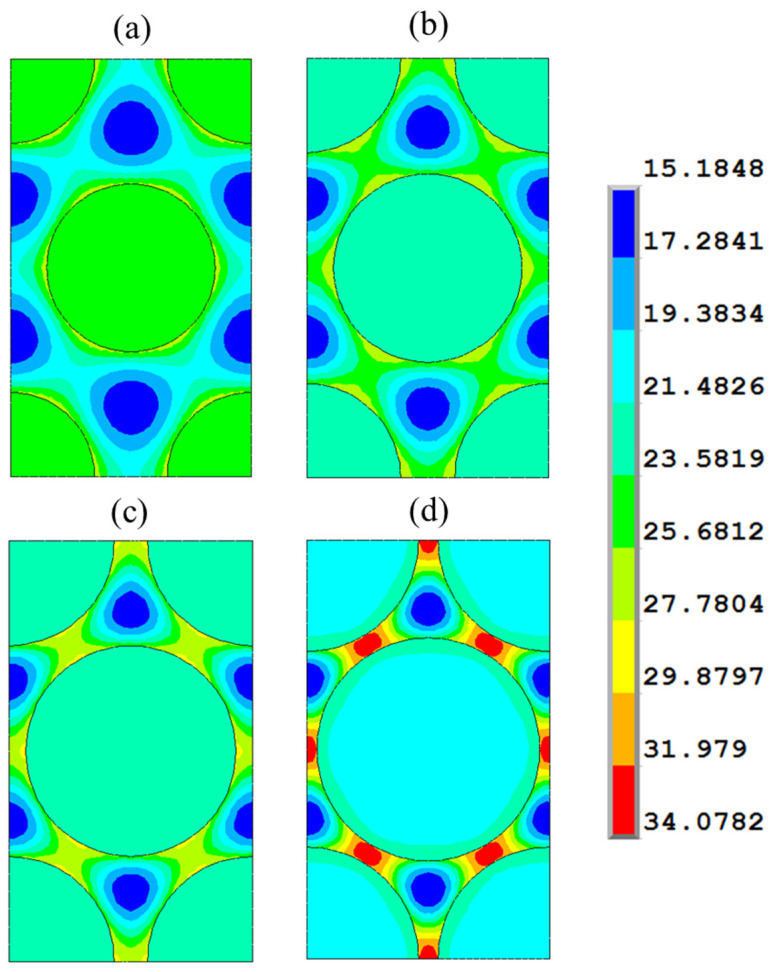
von Mises stress (MPa) contour plot in the hexagonal array RVE for different fiber fractions: (**a**) fiber fraction V_f_ = 0.4, (**b**) fiber fraction V_f_ = 0.5, (**c**) fiber fraction V_f_ = 0.6, and (**d**) fiber fraction V_f_ = 0.7.

Figure 8 illustrates the contour plots of the von Mises stresses for hexagonal configurations with different fiber fractions (V_f_ = 0.4, 0.5, 0.6, and 0.7), showing that the maximum stress in the resin increases with the fiber fraction.

**Figure 9:**
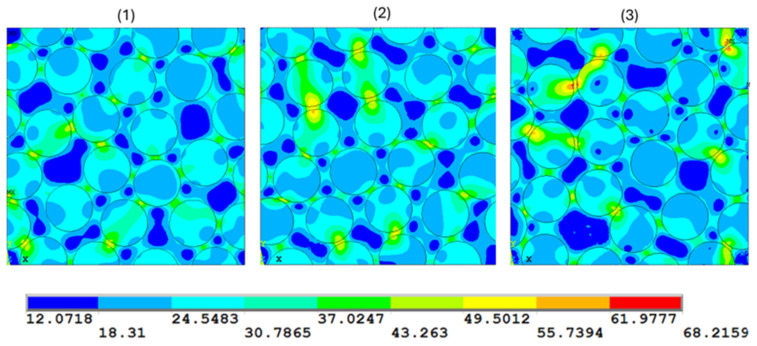
Von Mises stress (MPa) contour plot in three random configurations (fiber fraction V_f_ = 0.6).

Von Mises contour plots for the random arrays are shown in Figure 9. The stress intensity is higher in areas with a higher fiber fraction and lower in the matrix-rich regions. Since fibers and the matrix have very different thermal and mechanical properties, the fibers act as constraints that limit the deformation of the matrix due to free loads, and internal stresses arise because of these mechanical constraints. At various points, the residual stresses at the end of the process approach the nominal strength of the resin, meaning that the matrix is already significantly damaged in some areas at the end of the manufacturing process before applying any external mechanical loads.

**Figure 10:**
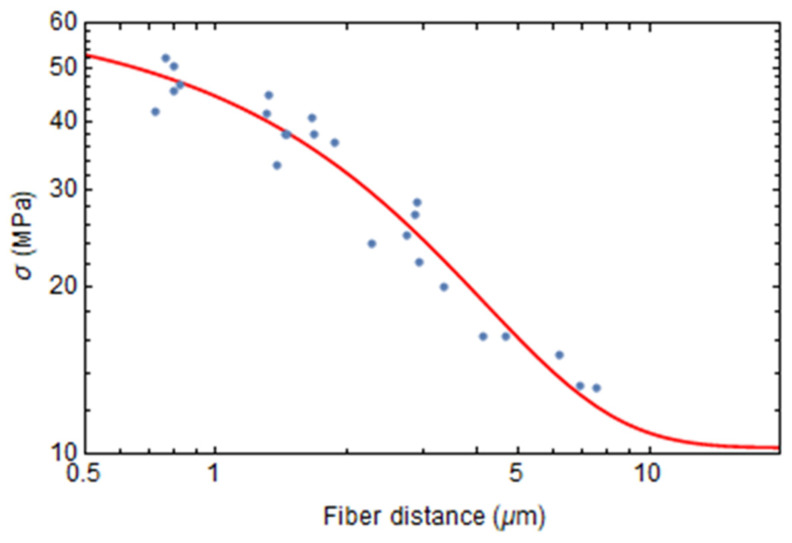
von Mises stress in the resin versus the distance between two fibers. Blue dots: numerical values. Red line: fitted interpolation curve.

Moreover, it is noted that the residual stresses in the portion of resin located between two adjacent fibers scale almost exponentially with the distance *x* separating two fibers, as shown in Figure 10. The numerical values of stresses at different distances are fitted using the following empirical equation:

(29) $$\sigma =c_{0}+c_{1}\;exp\;(-\frac{x}{c_{2}})$$

The terms *c*_0_, *c*_1_, and *c*_2_ in Equation (29) are material parameters obtained by minimizing the approximation error.

In addition, a comparison was made between ideal initially unstressed RVEs and curing-induced stressed RVEs in order to assess the impact of the manufacturing process on the mechanical properties of the composite. In particular, since the composite transversal properties are strongly influenced by the matrix’s mechanical behavior, transversal tension, compression, and out-of-plane shear tests were simulated for both the initially unstressed configuration (where the curing stresses are not considered) and on a curing-induced stressed configuration (where effects of residual stresses are accounted for).

In the fiber direction, mechanical properties are fiber-dominated, so it is assumed that curing damage does not significantly affect composite properties in that direction. However, composite properties are strongly correlated to matrix damages in the traversal direction.

Matrix failure was numerically simulated by assuming that the element’s failure occurs when the element’s Von Mises stress exceeds a critical value (assumed to be 70 MPa). The EKILL command from the Ansys library was used: when an element exceeds the critical stress value, it can no longer carry the load and is deactivated. Its modulus and stress components are set to zero in the next time steps. The NLGEOM option was activated to consider the non-linearity effects due to the stiffness reduction after element deactivation. Since the simulation of mechanical tests was performed at room temperature, an elastic behavior of the epoxy was assumed.

The tensile, compression, and shear tests were performed by applying displacement difference between the two opposite faces of the RVE, using periodic boundary conditions (Equations (24)–(27)).

**Figure 11:**
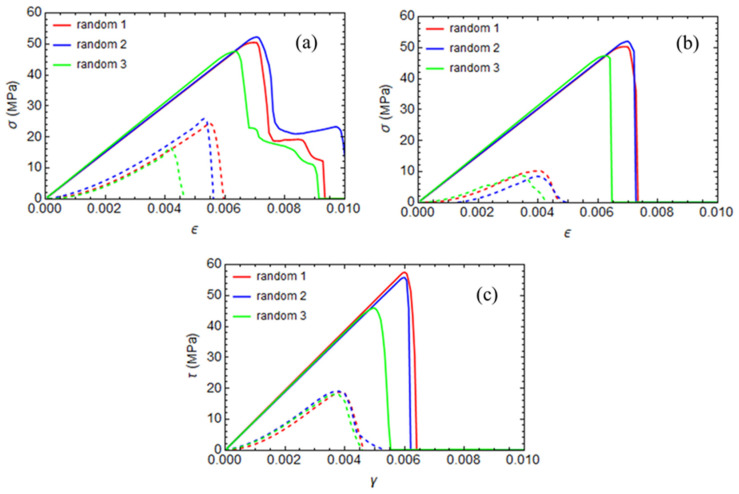
Stress–strain curves for the random RVEs. Continuous lines represent the initially unstressed configuration, and dashed lines represent the initially cure-stressed configuration. (**a**) Tension, (**b**) compression, and (**c**) shear.

**Figure 12:**
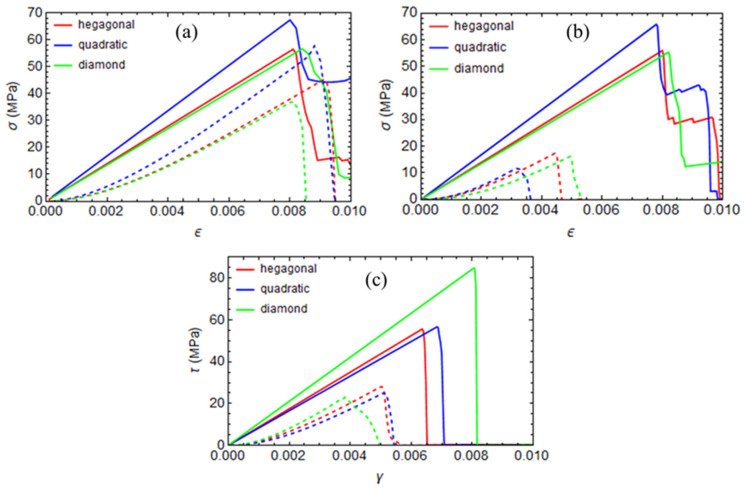
Stress–strain curves for the hexagonal, quadratic, and diamond RVEs. Continuous lines represent the initially unstressed configuration, and dashed lines represent the initially cure-stressed configuration. (**a**) Tension, (**b**) compression, and (**c**) shear.

The static analysis results are reported in Figure 11 and Figure 12, where stress–strain curves for unstressed and curing-induced stressed configurations are compared for all the fiber arrays analyzed in the previous section. The results indicate that curing-induced residual stresses significantly affect the transverse direction’s mechanical properties. In particular, cure-induced damage influences the compressive strength more than tensile and shear strength.

The arrangement of fibers also affects the composite’s ultimate strengths. Specifically, the quadratic array exhibits higher tensile and compressed strength than the hexagonal and diamond arrangements in the cure-unstressed configuration. The diamond array has a higher shear strength.

In the cure-induced stressed configuration, the random fiber arrangement has a lower tensile strength than the other geometrical configurations; the fiber arrangement does not significantly affect the shear and compressive strengths.

**Figure 13:**
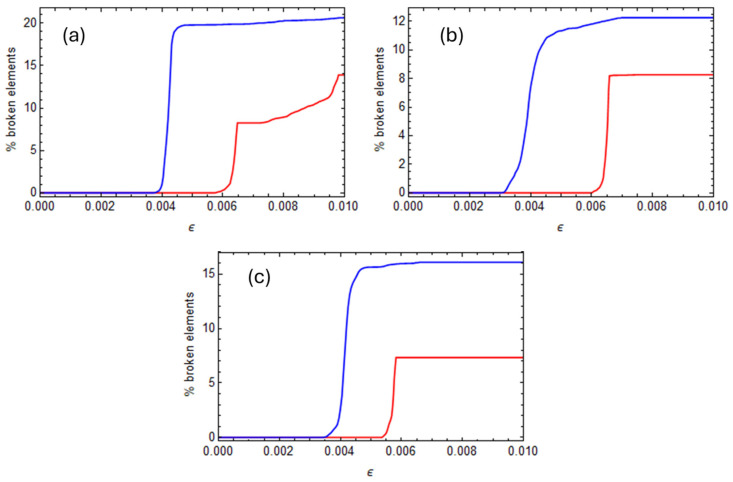
Percentage of matrix elements that failed in the random array during the loading phase as a function of the applied deformation. Red lines represent the initially unstressed configuration, and blue lines represent the cure-stressed configuration. (**a**) Tension, (**b**) compression, and (**c**) shear.

Related to a random array (representing the most realistic distribution), it is analyzed how the matrix elements fail when the loads are applied. In particular, Figure 13 reports the percentage of matrix elements that failed in the matrix during the loading phase as a function of the applied deformation for the tensile, compressive, and shear loads.

A comparison was made between the initially unstressed configuration (where the residual stresses are not considered) and a cure-stressed configuration (where residual stresses are included), for all considered load cases.

It can be observed that element failure begins just after the stress–strain curve reaches its peak. After that, failure occurs very fast in the matrix until the entire system is not able to sustain the applied load. Results show that, in the cure-induced stressed configuration, matrix failure initiates earlier, and a greater number of elements are broken at the end of the loading phase.

**Figure 14:**
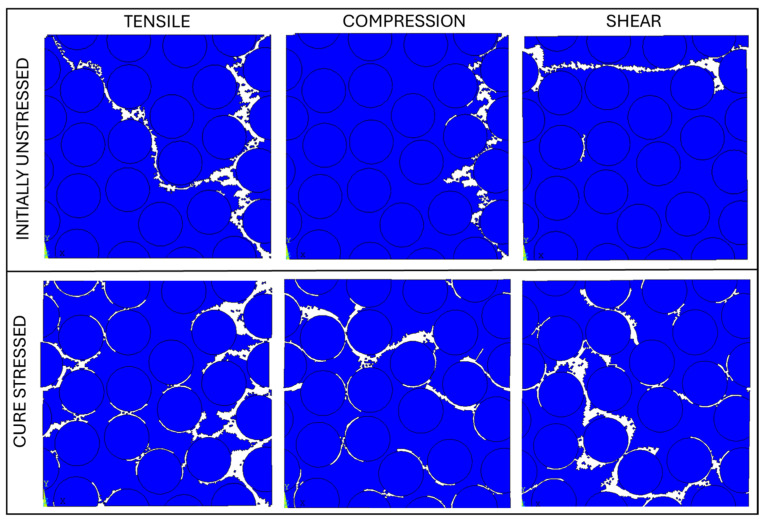
Distribution of broken elements at the end of loading for the random array. Comparison between the initially unstressed and cure-stressed configurations under different loading modes.

Figure 14 illustrates the final damage patterns in the random array for all the three load conditions.

It is evident that the cure-stressed configuration results in a higher percentage of failed matrix elements. In particular, in the initially unstressed configuration, damage tends to localize along a single main crack that propagates in the matrix until the structure fails (especially under compression and shear loads). Instead, the cure-stressed configuration exhibits a much broader spatial distribution of the damage. In this case, elements failure occurs in many areas at the interface between the fiber and the matrix, due to the complex stress distribution introduced during the curing process.

## 4. Conclusions

This article presented a numerical procedure to simulate the curing process’s effect on the composites’ mechanical properties. Specifically, it was applied to a carbon–epoxy composite at the micromechanical level to predict the development of residual stresses within the matrix during the composite cure. The results show that residual stresses especially arise during the cooling from curing to room temperature. These residual stresses constitute a significant fraction of the resin’s nominal strength.

Different fiber fractions and various geometric fiber distributions were analyzed. It was found that the stresses in the matrix phase increase with the fiber volume fraction.

Moreover, the fiber arrangement within the resin produces the same mean stress in the matrix but different stress distributions within the array. Specifically, it was found that the maximum von Mises stress in the quadratic and the diamond arrays is higher than in the hexagonal one. In random arrangements, the residual stresses are higher in fiber-rich zones and lower in fiber-poor areas. In some regions, their values approach the resin’s nominal strength.

Additionally, after curing, the RVEs were virtually tested in tensile, compression, and shear tests. These simulations evaluate the effects of curing-induced damage on the composite’s transverse mechanical properties by comparing the stress–strain curves with those of an ideal, initially unstressed material. It was noted that cure-induced stress significantly reduces the transversal mechanical properties, with a more significant effect on compressive loads compared to tensile and shear loads.

Future research should focus on extending this model from the microscale to the macroscale, evaluating also the effect of the stacking sequence and tool part interaction on the distortions and internal stresses arising in a composite part during the curing process.

## Figures and Tables

**Table 1:** Thermal and kinetic parameters for epoxy 3501.

A_1_	2.102 × 10^9^	min^−1^
A_2_	−2.014 × 10^9^	min^−1^
A_3_	1.960 × 10^5^	min^−1^
ΔE_1_	8.07 × 10^4^	J/mol
ΔE_2_	7.78 × 10^4^	J/mol
ΔE_3_	5.66 × 10^4^	J/mol
H_r_	473.16	kJ/Kg
R	8.314	J/Kg mol
ρ	1200	Kg/m^3^
c_p_	1260	J/Kg K
k	0.167	W/m K

**Table 2:** The Prony’s coefficients for the shear relaxation moduli at the reference state.

i	$\tau_{i}^{G}$ (s)	$\alpha_{i}^{G}$
1	1.75 × 10^−9^	0.059
2	1.75 × 10^−7^	0.066
3	1.09 × 10^−5^	0.083
4	6.60 × 10^−4^	0.112
5	1.70 × 10^−2^	0.154
6	4.76 × 10^−1^	0.262
7	1.17 × 10^1^	0.184
8	2.00 × 10^2^	0.049
9	2.95 × 10^4^	0.025

**Table 3:** Material parameters defining the glass transition temperature (*T_g_*), the activation energy (Δ*H*), and the non-linearity parameter (*x*).

b_1_	284.46
b_2_	−47.33
b_3_	239.4
k_4_	5.715 × 10^5^
k_5_	−5.10 × 10^5^
k_6_	1.92 × 10^5^
k_7_	0.833
k_8_	0.0374

**Table 4:** Prony’s coefficients of memory function at the reference state.

i	$\tau_{i}^{M}$ (s)	$\alpha_{i}^{M}$
1	1.21 × 10^−6^	0.0062
2	2.60 × 10^−5^	0.0072
3	5.60 × 10^−4^	0.0175
4	1.21 × 10^−2^	0.0390
5	2.60 × 10^−1^	0.0856
6	5.60	0.1730
7	1.21 × 10^2^	0.2950
8	2.60 × 10^3^	0.298
9	5.60 × 10^4^	0.0785

**Table 5:** Fibers’ mechanical properties.

E_11_	276	GPa
E_22_, E_33_	16.5	GPa
ν_12_, ν_13_	0.28	
ν_12_	0.7	
G_12_, G_13_	70	GPa
G_12_	5.7	GPa
CTE_1_	−0.54 × 10^−6^	
CTE_2_, CTE_3_	10.1 × 10^−6^	

## Data Availability

The raw data supporting the conclusions of this article will be made available by the authors on request.
